# The LF/HF ratio does not accurately measure cardiac sympatho-vagal balance

**DOI:** 10.3389/fphys.2013.00026

**Published:** 2013-02-20

**Authors:** George E. Billman

**Affiliations:** Department of Physiology and Cell Biology, The Ohio State UniversityColumbus, OH, USA

Power spectral analysis of the beat-to-beat variations of heart rate or the heart period (R–R interval) has become widely used to quantify cardiac autonomic regulation (Appel et al., 1989; Task Force of the European Society of Cardiology and the North American Society of Pacing and Electrophysiology, 1996; Berntson et al., 1997; Denver et al., 2007; Thayler et al., 2010; Billman, 2011). This technique partitions the total variance (the “power”) of a continuous series of beats into its frequency components, typically identifying two or three main peaks: Very Low Frequency (VLF) <0.04 Hz, Low Frequency (LF), 0.04–0.15 Hz, and High Frequency (HF) 0.15–0.4 Hz. It should be noted that the HF peak is shifted to a higher range (typically 0.24–1.04 Hz) in infants and during exercise (Berntson et al., 1997). The HF peak is widely believed to reflect cardiac parasympathetic nerve activity while the LF, although more complex, is often assumed to have a dominant sympathetic component (Task Force of the European Society of Cardiology and the North American Society of Pacing and Electrophysiology, 1996; Berntson et al., 1997; Billman, 2011). Based upon these assumptions, Pagani and co-workers proposed that the ratio of LF to HF (LF/HF) could be used to quantify the changing relationship between sympathetic and parasympathetic nerve activities (i.e., the sympatho-vagal balance) (Pagani et al., 1984, 1986; Malliani et al., 1991) in both health and disease. However, this concept has been challenged (Kingwell et al., 1994; Koh et al., 1994; Hopf et al., 1995; Eckberg, 1997; Houle and Billman, 1999; Billman, 2011). Despite serious and largely under-appreciated limitations, the LF/HF ratio has gained wide acceptance as a tool to assess cardiovascular autonomic regulation where increases in LF/HF are assumed to reflect a shift to “sympathetic dominance” and decreases in this index correspond to a “parasympathetic dominance.” Therefore, it is vital to provide a critical assessment of the assumptions upon which this concept is based.

The hypothesis that LF/HF accurately reflects sympatho-vagal balance rests upon several interrelated assumptions as follows (modified from Eckberg, 1997): (1) cardiac sympathetic nerve activity is a major, if not the exclusive, factor responsible for the LF peak of the heart rate power spectrum; (2) cardiac parasympathetic is exclusively responsible for the HF peak of the heart rate power spectrum; (3) disease or physiological challenges provoke reciprocal changes in cardiac sympathetic and parasympathetic nerve activity (i.e., increases in cardiac parasympathetic nerve activity are always accompanied with corresponding reductions in cardiac sympathetic nerve activity and *vice versa*); and (4) there is a simple linear interaction between the effects of cardiac sympathetic and cardiac parasympathetic nerve activity on heart rate variability (HRV).

As previously noted, frequency domain analysis of HRV usually reveals two or more peaks, a lower frequency (<015 Hz) and a higher frequency peak (>0.15 Hz) that are often assumed to correspond to cardiac sympathetic and cardiac parasympathetic neural activity, respectively (Pagani et al., 1984, 1986; Malliani et al., 1991). However, accumulating evidence clearly demonstrates that this assumption is naïve and greatly oversimplifies the complex non-linear interactions between the sympathetic and the parasympathetic divisions of the autonomic nervous system (Berntson et al., 1997; Eckberg, 1997; Parati et al., 2006; Billman, 2009, 2011). This is particularly true with regards to the relationship between LF power and cardiac sympathetic regulation (Randall et al., 1991; Ahmed et al., 1994; Kingwell et al., 1994; Hopf et al., 1995; Eckberg, 1997; Houle and Billman, 1999; Parati et al., 2006; Billman, 2009, 2011).

The LF peak of the heart rate power spectrum is reduced by at least 50% by either cholinergic antagonists or selective parasympathectomy (Akselrod et al., 1981; Randall et al., 1991; Houle and Billman, 1999). Importantly, this peak is not completely eliminated by the combination of selective denervation and beta-adrenoceptor blockade (Randall et al., 1991); ~25% of the peak remains after this treatment. As a consequence, LF/HF often actually increases from baseline values when both parasympathetic and adrenergic nerve activity have been blocked. For example, using the data reported by Randall and co-workers (Randall et al., 1991), LF/HF increased from a baseline value of 1.1–8.4 when selective parasympathetic denervation was combined with beta-adrenergic receptor blockade, falsely suggesting a major shift to sympathetic dominance! In a similar manner, interventions that would be expected to increase cardiac sympathetic activity, such as acute exercise or myocardial ischemia, not only failed to increase LF power but actually provoked significant reductions in this variable (Houle and Billman, 1999), once again yielding LF/HF values that are difficult to interpret. Indeed, despite large increases in heart rate, LF/HF ratio was largely unaffected by either acute myocardial ischemia, exercise, or the cholinergic antagonist atropine sulfate (Houle and Billman, 1999). Finally, direct recording of sympathetic nerve activity failed to correlate with LF power in either healthy subjects or patients with heart failure (Hopf et al., 1995; Notarius and Floras, 2001; Jardine et al., 2002; Moak et al., 2007; Piccirillo et al., 2009), a condition known to increase cardiac sympathetic drive (Hasking et al., 1986; Saul et al., 1988; Watson et al., 2007). Thus, the LF component of HRV does not provide an index of cardiac sympathetic drive but rather reflects a complex and not easily discernible mix of sympathetic, parasympathetic, and other unidentified factors with parasympathetic factors accounting for the largest portion of the variability in this frequency range. As a consequence, the physiological basis for LF/HF is difficult to discern.

Although the vast majority of the clinical and the experimental studies demonstrate a strong association between HF power and cardiac parasympathetic activity (Katona et al., 1970; Appel et al., 1989; Billman and Hoskins, 1989; Billman and Dujardin, 1990; Task Force of the European Society of Cardiology and the North American Society of Pacing and Electrophysiology, 1996; Billman, 2009, 2011; Thayler et al., 2010), this concept has also been challenged (Kollai and Mizsei, 1990; Goldberger et al., 1994; Hedman et al., 1995; Taylor et al., 2001; Parati et al., 2006). Unlike LF power and sympathetic nerve activity, a strong correlation between HF power and direct recordings of cardiac parasympathetic activity has been reported (Chess et al., 1975; Piccirillo et al., 2009). However, just as parasympathetic activation exerts profound influences on the LF component of HRV, sympathetic neural activity may modulate the HF component of the R–R interval variability (Taylor et al., 2001; Cohen and Taylor, 2002). Taylor et al. (2001) found that cardioselective beta-adrenergic receptor blockade (drugs that should not indirectly alter vagal outflow via action within the central nervous system) increased the amplitude of the respiratory sinus arrhythmia over a wide range of respiratory frequencies (i.e., the increases were not restricted to lower frequencies, <0.15 Hz). They concluded that “*cardiac sympathetic outflow can oppose vagally mediated R-R interval oscillations and sympathetic blockade removes this effect*” (Cohen and Taylor, 2002). Based upon these data, sympathetic nerve activation may alter the HF peak by perhaps as much as 10%. Thus, differences in cardiac sympathetic activation during a physiological challenge (e.g., exercise or postural changes) in healthy subjects or that occur as the consequence of cardiovascular disease (following myocardial infarction) could restrain vagally mediated changes in HRV. These data suggest that HF power cannot be solely attributed to changes in cardiac vagal efferent nerve traffic, further compromising an accurate interpretation of the LF/HF ratio.

Accurate interpretation of LF/HF ratio also depends upon the assumption that physiological interventions always elicit reciprocal changes in parasympathetic and sympathetic nerve activity. However, following the termination of exercise sympathetic activation remains high despite the rapid re-activation of cardiac parasympathetic drive (Smith et al., 2005; Billman and Kukielka, 2007; Billman, 2009). Furthermore, chemoreceptor activation by carbon dioxide provokes parallel reductions in sympathetic and parasympathetic nerve activity (Eckberg, 1997) while facial emersion in cold water (activating the so-called “diving reflex”) increased sympathetic nerve activity yet elicited a profound bradycardia (Eckberg et al., 1984; Fagius and Sundlof, 1986). The observation that heart rate declines, despite increases in sympathetic nerve activity, highlights the complex non-linear interactions of the sympathetic and parasympathetic nervous system, providing an example of “accentuated antagonism” (Levy, 1971; Stramba-Badiale et al., 1991; Uijtdehaage and Thayer, 2000), the dominance of parasympathetic over sympathetic influences on cardiac rate. Finally, reciprocal changes in parasympathetic and sympathetic nerve activity do not always occur even during the activation of the baroreceptor reflex (Eckberg, 1997). Eckberg and co-workers have shown that, although small changes in arterial pressure typically provoke reciprocal changes sympathetic and parasympathetic nerve activity, large increases in arterial pressure only provoke increases in parasympathetic nerve activity without altering the prevailing sympathetic activity (Eckberg, 1980; Rea and Eckberg, 1987). Furthermore, autonomic response to baroreceptor reflex activation depends on whether the pressure changes occur near the threshold or the saturation point of the response curve; the same change in pressure can elicit larger or smaller autonomic responses depending on how close the prevailing pressure lies to the threshold (larger) or saturation (smaller) portion of the stimulus-response curve (Eckberg, 1980, 1997). As previously noted, changes in heart rate do not result from the simple algebraic summation of the sympathetic and parasympathetic nerve activity. Rather, parasympathetic nerve activation can completely override even maximal sympathetic nerve stimulation, provoking large reductions in heart rate in the face of sympathetic nerve activation as was previously noted for the diving reflex. Thus, physiological interventions can elicit either complex non-linear reciprocal or parallel changes in either division of the autonomic nervous system. These complex interactions can profoundly influence the calculation and the interpretation of LF/HF.

**Figure 1:**
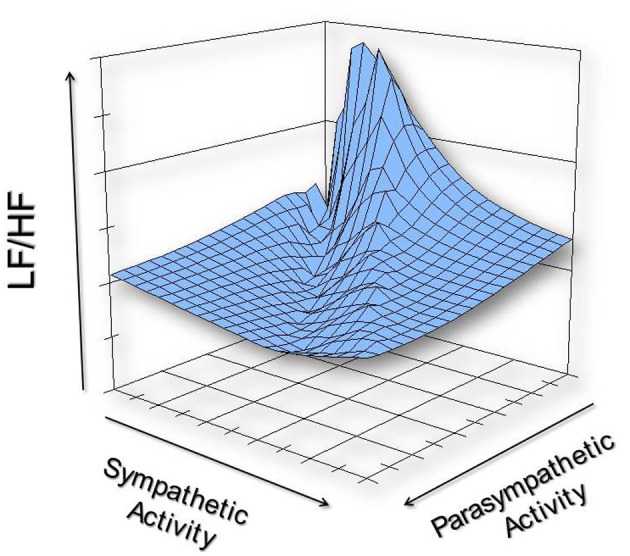
**An illustration of the possible non-linear effects of varying cardiac sympathetic and cardiac parasympathetic nerve activity on LF/HF.** This graph was constructed using the following formula (derived from a synthesis of the literature, particularly Randall et al., 1991; Taylor et al., 2001; Cohen and Taylor, 2002): LF/HF = (0.5 parasympathetic + 0.25 sympathetic nerve activity)/(0.9 parasympathetic + 0.1 sympathetic nerve activity). The nerve activity was varied from baseline (1 arbitrary unit each) increasing or decreasing by up to a factor of 10 (i.e., from 0.1 to 10 units).

Mathematical considerations can also influence LF/HF values. Similar LF/HF values can be obtained via either exclusive changes in the numerator (i.e., LF), or the dominator (i.e., HF), or by some combination of the two, as is illustrated in Table 1. For example, a doubling of parasympathetic activity against maintained sympathetic nerve activation yields the identical LF/HF value as a 50% reduction in sympathetic nerve activity against a constant background parasympathetic regulation. Based upon the literature, one can conclude that parasympathetic nerve activation contributes to at least 50% of the LF variability while sympathetic activity, at best, only contributes 25% to this variability (Randall et al., 1991). A substantial portion of the variability in the LF band also results from other unidentified factors. In a similar fashion, sympathetic nerve activity could contribute to perhaps as much as 10% of the HF variability (Taylor et al., 2001; Cohen and Taylor, 2002). As a consequence, the effects of changing sympathetic and parasympathetic activity on the LF/HF are quite variable and not intuitively obvious, as is illustrated in Figure 1. This figure was constructed using the following formula that was based upon a synthesis of the literature (particularly, Randall et al., 1991; Taylor et al., 2001; Cohen and Taylor, 2002), LF = 0.5 parasympathetic + 0.25 sympathetic activity while HF = 0.9 parasympathetic + 0.1 sympathetic nerve activity. The nerve activity was varied from baseline (1 arbitrary unit each) increasing or decreasing by up to a factor of 10. Due to the substantial contribution (accounting for up to 25% of the variability) (Randall et al., 1991) from non-neural factors to LF power, very distorted values of LF/HF can be obtained when both sympathetic and parasympathetic nerve activity are minimal. If for example, one assumes that LF = 0.5 parasympathetic + 0.25 sympathetic + 0.25 other factors and both parasympathetic and sympathetic nerve activity are reduced to 1/100 the baseline values, the calculated LF/HF becomes (0.005 + 0.0025 + 0.25)/(0.009 + 0.001) = 25.75! Despite the almost complete absence of cardiac autonomic regulation, this value could be inappropriately interpreted as a major shift toward sympathetic dominance. Furthermore, LF/HF cannot be determined if both sympathetic activity and parasympathetic nerve activity were to be abolished completely (i.e., when the dominator is zero). Finally, mathematical complications also arise due to the non-linear relationship between R–R interval and heart rate; similar changes in heart rate elicit much greater variability in R–R interval at lower than at higher heart rates (Sacha and Pluta, 2008). As a consequence of this non-linear relationship, it is difficult to separate the changes in HRV that arise from direct action of cardiac autonomic nerves from those changes that result indirectly from neurally induced changes in average heart rate. This observation led Sacha and Pluta (2008), to propose that HRV has both physiological and mathematical influences that can be corrected by the division of HRV by average R–R interval. Thus, the physiological basis for changes in LF/HF is not readily discernible and spurious values for LF/HF can result as a consequence of the mathematical manipulations of the data.

**Table 1:** **Examples of the effects of varying cardiac sympathetic and parasympathetic nerve activity on LF/HF**.

**Parasympathetic nerve activity**	**Sympathetic nerve activity**	**LF**	**HF**	**LF/HF**
1	1	0.75	1	0.75
2	1	1.25	1.9	0.66
0.5	1	0.5	0.55	0.91
1	2	1	1.1	0.91
1	0.5	0.625	0.95	0.66
2	2	1.5	2	0.75
2	0.5	1.125	1.85	0.61
0.5	2	0.75	0.65	1.15
0.5	0.5	0.375	0.5	0.75

These numbers were generated using the following formula (derived from a synthesis of the literature, particularly Randall et al., 1991; Taylor et al., 2001; Cohen and Taylor, 2002): LF/HF = (0.5 parasympathetic + 0.25 sympathetic nerve activity)/(0.9 parasympathetic + 0.1 sympathetic nerve activity). The nerve activity is reported as arbitrary units where at baseline sympathetic and parasympathetic nerve activity were normalized as 1 arbitrary unit each. The data shown are for baseline and various combinations of doubling (2 × baseline) or halving (0.5 × baseline) the autonomic nerve activity.

It should also be noted, that HRV (and thereby LF/HF) is affected by respiratory parameters and mechanical events independent of changes in cardiac autonomic nerve activity. The contribution of mechanical factors (due to stretch of the atria that results from both changes in cardiac filling and the changing thoracic pressure that occur during respiration) to changes in HRV was first proposed by Bainbridge (1930). This conclusion is supported by the observation that heart transplant patients, despite the absence of cardiac nerves, still exhibit small (~2–8% of normal) change in R–R interval associated with the respiratory cycle (Bernardi et al., 1989). Taylor et al. (2001) further demonstrated that atrial stretch can exert significant influences on R–R interval in subjects with complete autonomic blockade. They found that after combined cholinergic and adrenergic receptor blockade slow deep breathing could still provoke oscillations of ~120 ms in healthy human subjects (Taylor et al., 2001). In similar manner, mechanical distortion (stretch) of the sinoatrial nodal stretch in pigs without functional autonomic innervation (vagal nerve section combined with propranolol treatment) reduced the HF component of HRV (Horner et al., 1996).

Respiratory parameters can also profoundly alter heart rate and R–R interval variability independent of changes in cardiac autonomic regulation (i.e., against a constant background level of automatic regulation) (Angelone and Coulter, 1964; Davies and Neilson, 1967; Hainsworth, 1974; Melcher, 1976; Hirsch and Bishop, 1981; Brown et al., 1993; Van De Borne et al., 2001). It is now well established that increases in respiratory frequency reduce the amplitude of heart rate oscillations (Angelone and Coulter, 1964; Melcher, 1976; Hirsch and Bishop, 1981; Brown et al., 1993) while either increases in tidal (Davies and Neilson, 1967; Melcher, 1976; Hirsch and Bishop, 1981; Eckberg, 1983; Kollai and Mizsei, 1990; Brown et al., 1993) or static lung volume (Hainsworth, 1974) provoke increases in the R–R interval variability. The facts are in direct opposiiton to the assumptions. Conversely, reductions in respiratory frequency increase HRV (Angelone and Coulter, 1964; Melcher, 1976; Hirsch and Bishop, 1981; Brown et al., 1993) while decreases in tidal volume lead to reductions in the R–R interval variability (Davies and Neilson, 1967; Melcher, 1976; Hirsch and Bishop, 1981; Eckberg, 1983; Kollai and Mizsei, 1990; Brown et al., 1993). Thus, it is critical to control breathing (paced or timed breathing) in order to interpret HRV data accurately. For obvious reasons, it is much more difficult to control respiratory parameters in conscious animal than in human studies. However, these respiratory parameters frequently are not controlled even in human studies (Brown et al., 1993). Brown and co-workers (Brown et al., 1993), reviewed the human literature and found that only about 51% controlled respiratory rate, and even fewer studies controlled for tidal volume (11%). They further reported that respiratory parameters not only altered HF power but also strongly influenced the LF components of the R–R interval power spectrum, a component that previously was viewed to vary independently of changes in respiration (Brown et al., 1993).

Finally, prevailing heart rate can also influence HRV. There are a number of studies that report a strong positive correlation between mean R–R interval and various time domain indices of HRV (e.g., the standard deviation of normal beats, SDNN) (Kleiger et al., 1987; Van Hoogenhuyze et al., 1991; Fleiss et al., 1992) such that HRV was greater during longer mean R–R intervals (slower heart rates) than at shorter mean R–R intervals (faster heart rates). Frequency domain analysis of HRV is similarly affected by mean heart rate. Sacha and Pluta (2005) found that LF was directly related, while HF was indirectly related, to the average heart rate of the subject. As a consequence, they further report that LF/HF varied depending on heart rate, lower at slower and higher at faster heart rates. Thus, heart rate *per se* can influence LF/HF independent of changes cardiac autonomic nerve activity.

As we have seen, the hypothesis that LF/HF quantifies “sympatho-vagal balance” depends upon four interrelated assumptions, all of which can be proven to be false. The facts are in direct opposition to the assumptions. In particular, the complex nature of LF power, its exceedingly poor relationship to sympathetic nerve activation, and the non-linear (and often non-reciprocal) interactions between sympathetic and parasympathetic nerve activity that are confounded by the mechanical effects of respiration and prevailing heart rate, make it impossible to delineate the physiological basis for LF/HF with any degree of certainty. Thus, the LF/HF sympatho-vagal balance hypothesis has been disproven—the preponderance of evidence confirms that LF/HF data cannot accurately quantify cardiac “sympatho-vagal balance” either in health or disease.
